# Transcriptomic profiling reveals the dynamics of fibrotic progression‐related gene expression into post‐coronavirus disease 2019 pulmonary fibrosis

**DOI:** 10.1002/ctm2.70088

**Published:** 2024-11-13

**Authors:** Sabrina Setembre Batah, Andrea Jazel Rodriguez‐Herrera, Maria Júlia Faci do Marco, Juliana Rocha Souza Chiappetto, Mariana Gatto, Simone Alves do Vale, Robson Aparecido Prudente, Amanda Piveta Schnepper, Robson Francisco Carvalho, João Paulo Facio Almeida, Tales Rubens de Nadai, Marcel Konigkam Santos, Li Siyuan Wada, José Baddini‐Martinez, Danilo Tadao Wada, Andrea Antunes Cetlin, Vera Luiza Capelozzi, Bruno Guedes Baldi, Suzana Tanni, Rosane Duarte Achcar, Alexandre Todorovic Fabro

**Affiliations:** ^1^ Department of Pathology and Legal Medicine Ribeirão Preto Medical School University of São Paulo Ribeirao Preto Brazil; ^2^ Department of Internal Medicine Botucatu Medical School São Paulo State University Botucatu Brazil; ^3^ Institute of Biosciences São Paulo State University Botucatu Brazil; ^4^ Department of Biochemistry and Immunology Ribeirão Preto Medical School University of São Paulo Ribeirao Preto Brazil; ^5^ Bauru Medical School University of Sao Paulo Bauru Brazil; ^6^ Department of Surgery and Anatomy Ribeirão Preto Medical School University of São Paulo Ribeirao Preto Brazil; ^7^ Discipline of Pulmonology, São Paulo Medical School Federal University of São Paulo Sao Paulo Brazil; ^8^ Department of Medical Images Hematology and Oncology Ribeirão Preto Medical School University of São Paulo Ribeirao Preto Brazil; ^9^ Department of Internal Medicine Pulmonary Division Ribeirão Preto Medical School University of São Paulo Ribeirao Preto Brazil; ^10^ Department of Pathology, Faculty of Medicine University of São Paulo Sao Paulo Brazil; ^11^ Division of Pulmonology Heart Institute ‐InCor – Clinical Hospital Faculty of Medicine University of São Paulo Sao Paulo Brazil; ^12^ Department of Medicine National Jewish Health Pathology Division Denver Colorado USA

Dear Editor,

Our study has identified a gene expression profile associated with the progression of coronavirus disease 2019 (COVID‐19) to pulmonary fibrosis in a pro‐fibrotic environment similar to that found in fibrosing interstitial lung diseases (f‐ILDs). Briefly, we noted the common expression of 86 genes in post‐COVID‐19 pulmonary fibrosis (post‐CPF) and f‐ILDs, indicating their likely involvement in perpetuating pulmonary fibrosis through shared fibrotic pathways—confirmed by the in‐situ expression of MUC5ac and WNT10a. Furthermore, an additional set of 31 genes exhibited common expression patterns between subacute COVID‐19, the so‐called organizing diffuse alveolar damage (ODAD), and CPF, as well as f‐ILDs. Among those genes, MUC4 and KRT5 were confirmed by immunohistochemistry, suggesting their role as potential predictors for the early outcome of possible pulmonary fibrosis.

Post‐CPF is a long‐term complication diagnosed by clinical setting, pulmonary function tests and/or image examinations.^1^ Initially, some COVID‐19‐infected patients develop acute respiratory distress syndrome (ARDS) during the exudative phase of DAD, marked by cytokine storm and immune cell recruitment.^2^ Following the inflammatory peak and pneumocyte injury, myofibroblast activation triggers extracellular matrix (ECM) deposition, leading to ODAD‐phase which typically restores to typical lung architecture. However, some patients progress to pulmonary fibrosis^3^ with morphological changes that are driven by a complex pathophysiological sequence and dynamic gene expression shifts. In the end, the fibrotic outcome can resemble other f‐ILDs. Identifying gene expression levels linked to the progression from ODAD to CPF is crucial for finding biomarkers for early diagnosis. Our study aimed to identify potential biomarkers in gene expression associated with fibrotic progression to CPF by analyzing the transcriptome of patients with ODAD, CPF, f‐ILDs and controls.

As previously described by Batah et al.,^4^ autopsies from the ODAD group revealed ODAD‐phase with bronchiolar metaplasia, myxoid fibrosis, myofibroblastic activation and extensive alveolar septal thickening with collagen types I and III deposition (Table S1; Figure 1A–C). Meanwhile, after an average of 324.6 days following the initial positive nasopharyngeal swab for severe acute respiratory syndrome coronavirus 2 (SARS‐CoV‐2), patients from the CPF group developed pulmonary fibrosis with bronchiolar metaplasia and parenchymal remodelling with increased collagen deposition, especially type I (suppinfo1; Figure 1D–F). Although the common remodelling profile, the differential gene expression (DGE) analysis between ODAD and CPF revealed distinct gene signatures (Figure S1A,B), Some of the top 20 DGEs reflect the manifestation of ARDS in ODAD patients (Figure S1C,D and Table S2). For example, the upregulation of SERPINE1 ‐fibrinolysis inhibitor‐ in ODAD suggests the accumulation of deposited fibrin as a better pathophysiological response to SARS‐CoV‐2.^5^ However, as mentioned earlier, both groups revealed parenchymal remodelling, and the WP Lung Fibrosis gene set (GSEA systematic name M39477) highlighted only a few DGE between the groups: upregulated MMP9 and TERT and downregulated MUC5B and FGF1 in ODAD compared to CPF (Figure S1E and Table S3). Therefore, some fibrotic pathways might be present in both groups, suggesting a possible similarity with the transcriptomic profile of f‐ILD patients.

**FIGURE 1:**
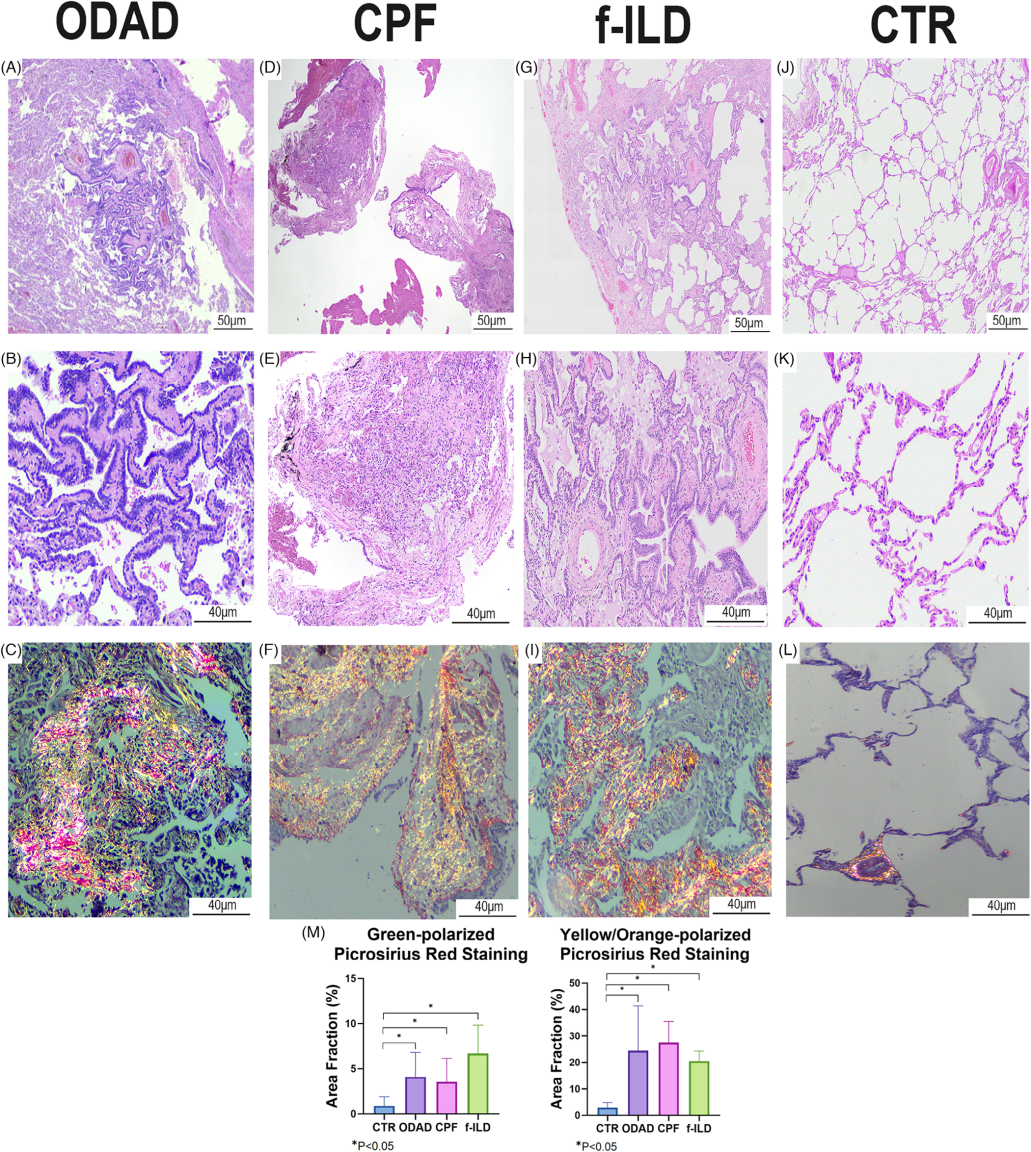
Histological panel of organizing diffuse alveolar damage (ODAD), coronavirus disease 2019 pulmonary fibrosis (CPF), fibrosing interstitial lung disease (f‐ILD) and CTR groups. ODAD group presented interstitial septal thickening with bronchiolar metaplasia of the epithelial cells (A, B) and intense collagen fibre deposition highlighted by Picrosirius Red staining (C); CPF group presented diffuse interstitial septal thickening (D, E) with increased collagen fibre deposition evidenced by Picrosirius Red staining (F); f‐ILD group presented predominantly bronchiolocentric fibrosis with bronchiolar metaplasia of the epithelial cells (G, H) and collagen fibre deposition emphasized by Picrosirius Red staining (I); CTR group presented typical lung architecture without any sign of interstitial septal thickening (J, K) showing minimal collagen fibre deposition marked by Picrosirius Red staining (L). The morphometric analysis of Picrosirius Red staining revealed that the CTR group exhibited significantly less deposition of collagen fibres compared to the ODAD, CPF, and f‐ILD groups (M).

In fact, our data revealed a significant similarity in gene expression among the groups compared to f‐ILD, presenting dense fibrosis, architectural distortion, bronchiolar metaplasia, fibroblast foci and collagen types I and III deposition (Table S1 and Figure 1G–I). The similarity is seen by the overlap of cases in the paired DGE analyses between CPF versus f‐ILD (Figure S2A,B) and ODAD versus f‐ILD (Figure S3A,B). In both principal component analyses, f‐ILD patients were divided into two distinct clusters: In Figure S2A, Cluster A is distinct from the other cases, while Cluster B is found among the CPF patients. Cluster A displays a bronchiolocentric remodelling pattern, whereas Cluster B exhibits a non‐specific lesion pattern characterized by uniform thickening of the pulmonary interstitium, similar to that observed in CPF patients. Similarly, in Figure S3A, Cluster D has the same patients as Cluster A, who also exhibit bronchiolocentric ECM deposition; while Cluster E has a lesion pattern more similar to ODAD, which is also non‐specific. This may suggest that the transcriptomic profiles of each cluster differently influence the pattern and intensity of ECM deposition, resulting in distinct forms of fibrosis depending on the genes expressed.

In addition, the top 20 DGE highlighted upregulation of COL1A1 and CDH23 in CPF versus f‐ILD (Figure S2C,D and Table S4); and downregulation of miR‐663a in ODAD versus f‐ILD, which directly targets transforming growth factor (TGF)‐β1^6^ (Figure S3C‐D and Table S5). However, the WP Lung Fibrosis gene set (GSEA systematic name M39477) did not reveal DGE between the groups, reinforcing the similarity in the fibrotic pathway pattern (Figures S2E and S3E; Tables S6 and S7).

To ascertain the similarity in the gene expression levels of CPF and ODAD with f‐ILD, we performed the DE analysis of each group against CTR‐minimally altered pulmonary parenchyma with significantly lower collagen fibre deposition compared to the other groups (Table S1 and Figure 1J–M). A Venn diagram was generated from the DGE results (ODAD vs. CTR, CPF vs. CTR and f‐ILD vs. CTR) to highlight the overlapping up and downregulated genes. As a result, 86 genes (71 upregulated, 15 downregulated) were commonly expressed in CPF and f‐ILD (Table S8 and Figure 2). The distinctiveness of these genes lies in their absence from the ODAD group, suggesting transcription under chronic injury conditions without an active injurious stimulus, which might be considered as “fibrosis‐related genes”. These genes likely play a pivotal role in sustaining fibrosis in a chronic and advanced state.

**FIGURE 2:**
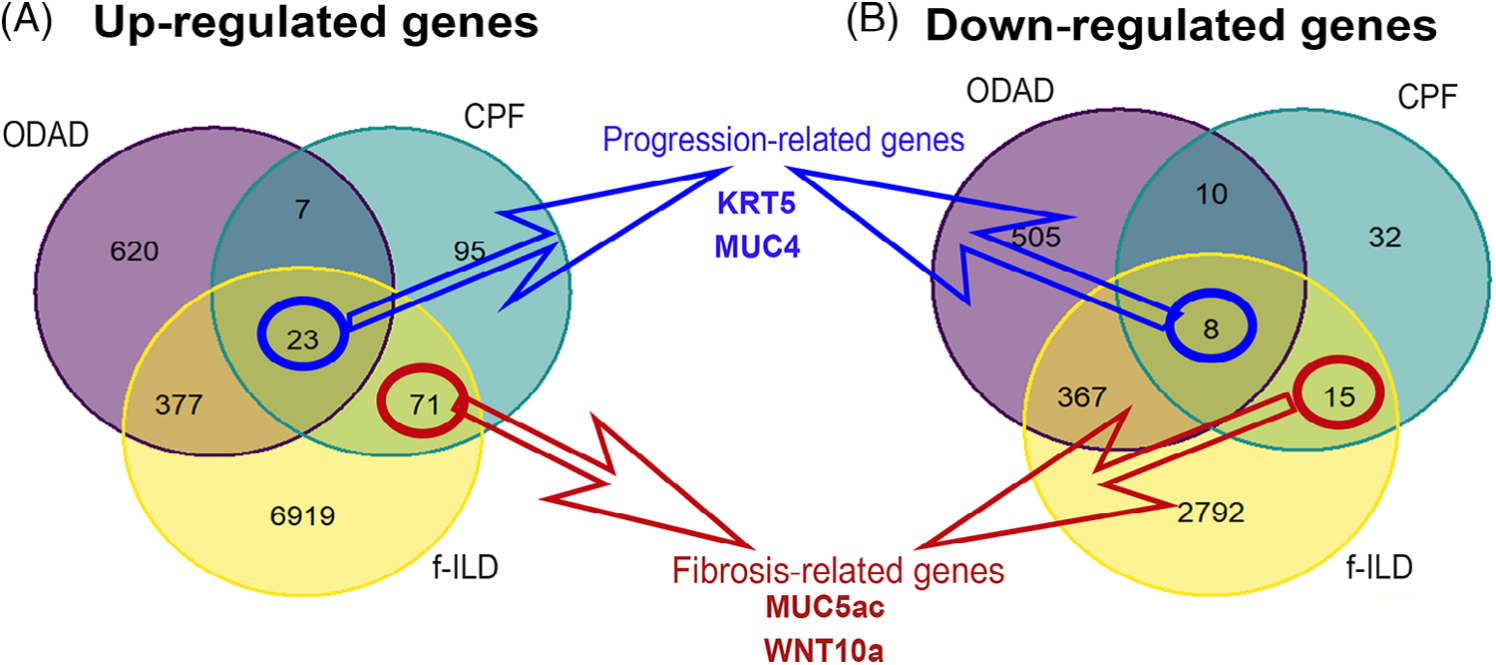
Venn diagram revealed an overlap of differentially expressed genes. The differentially expressed genes resulting from the DE analysis between organizing diffuse alveolar damage (ODAD), COVID‐19 pulmonary fibrosis (CPF), and fibrosing interstitial lung disease (f‐ILD) versus CTR group were plotted on a Venn diagram to emphasize the overlaps between the groups. There are 23 commonly upregulated genes and eight commonly downregulated genes among ODAD (purple), CPF (light blue) and f‐ILD (yellow), representing the progression‐related genes (A, B). Additionally, there are 71 commonly upregulated genes and 15 commonly downregulated genes between CPF (light blue) and f‐ILD (yellow), representing the fibrosis‐related genes (A, B).

Indeed, the upregulated Fibrosis‐related genes show significant enrichment in pathways related to WNT signalling (Figure 3A,B), suggesting a potential role in the progression of post‐CPF, possibly by promoting fibrosis and epithelial‐mesenchymal transition (EMT).^7^ Additionally, the Cadherin signalling pathway (Figure 3B) is also significantly enriched, further supporting its involvement in EMTs. To confirm the gene expression findings (Figure S4A–D), we selected two genes from the group of 86, MUC5ac and WNT10a, both of which are already widely used in diagnostic immunohistochemistry (IHC). We observed higher expression of goblet cell hyperplasia MUC5ac+ in CPF and f‐ILD compared to the other groups (Figure 3C,D, *p* < .05), as reported in other studies^8^; and higher in‐situ presence of WNT10a+ in squamous cell metaplasia in CPF and f‐ILD (Figure 3E,F, *p* < .05), consistent with its role in pulmonary fibrosis as described in other studies.^7^

**FIGURE 3:**
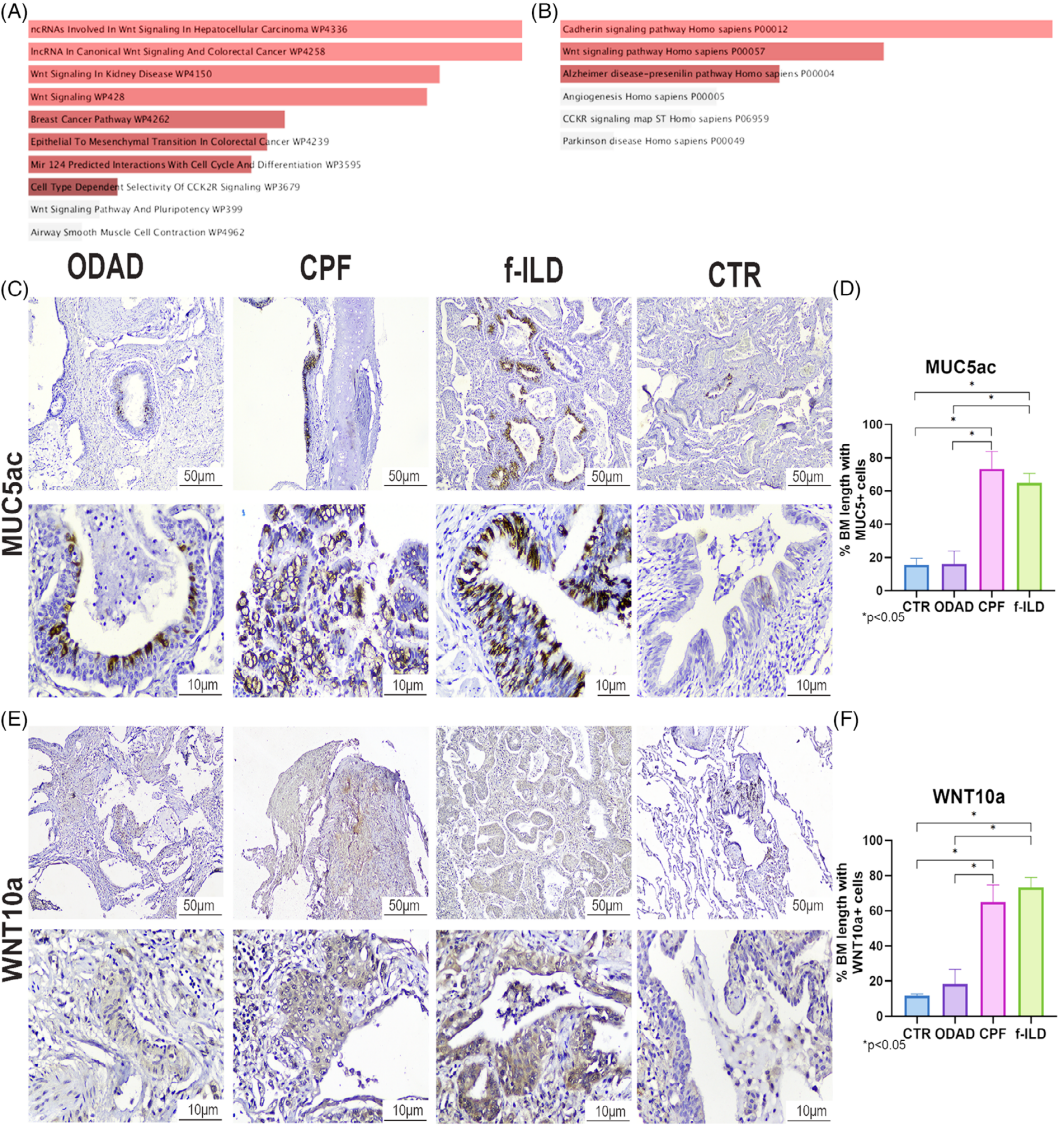
Enrichment analysis and immunohistochemistry of fibrosis‐related genes. The upregulated fibrosis‐related genes show significant enrichment in pathways related to WNT signalling: “ncRNAs involved in Wnt signalling in hepatocellular carcinoma WPA336” *p*‐value = .003438, *p*_adj_ = .09726; “lncRNA in canonical Wnt signalling and colorectal cancer WP4258” *p*‐value = .004427, *p*_adj_ = .09726; “Wnt signalling In Kidney Diseases WP4150” *p*‐value = .007241, *p*_adj_ = .09726; “Wnt signalling WP428” *p*‐value = .007781, *p*_adj_ = .09726 (A); “Wnt signalling pathway Homo sapiens P00057” *p*‐value = .01715, *p*_adj_ = .05145 (B); and significant enrichment in “Cadherin signalling pathway homosapiens P00012” p‐value = .002000, *p*_adj_ = .01200 (B). Immunohistochemistry revealed a marked goblet cell hyperplasia with MUC5ac+ expression in response to injurious stimulation within a fibrotic environment (C). This increased expression was observed in coronavirus disease 2019 (COVID‐19) pulmonary fibrosis (CPF) and fibrosing interstitial lung disease (f‐ILD) compared to organizing diffuse alveolar damage (ODAD) and CTR (*p* < .05) (D). Bronchiolar epithelial cells in squamous metaplasia exhibited WNT10a expression (E) in greater quantity in the CPF and f‐ILD groups compared to ODAD and CTR (*p* < .05) (F). *Note: BM: basement membrane.

The Venn diagram also emphasized 31 genes (23 upregulated and eight downregulated) commonly expressed across the three groups (ODAD, CPF and f‐ILD) (Table S9 and Figure 2). These genes likely contribute to the transition from the early fibrotic stage in ODAD to the more chronic fibrotic injury observed in CPF. Their continued presence in well‐established fibrosis (f‐ILD) underscores their importance in fibrosis progression, leading us to classify them as “progression‐related genes.” The enrichment analysis of the upregulated genes from this list revealed a significant association with the transcription factors SMAD2 and SMAD3 (Figure 4A), which are central to TGF‐β signalling, a key pathway in EMT and the advancement of fibrotic diseases, including post‐CPF. Additionally, these genes showed a strong link to the “innate immunity evasion and cell‐specific immune response” pathway associated with SARS‐CoV‐2 infections (Figure 4B). This pathway is crucial for understanding how the virus evades the host immune responses, potentially influencing the development of severe conditions like post‐CPF by disrupting immune detection and altering cell cycle regulation.

**FIGURE 4:**
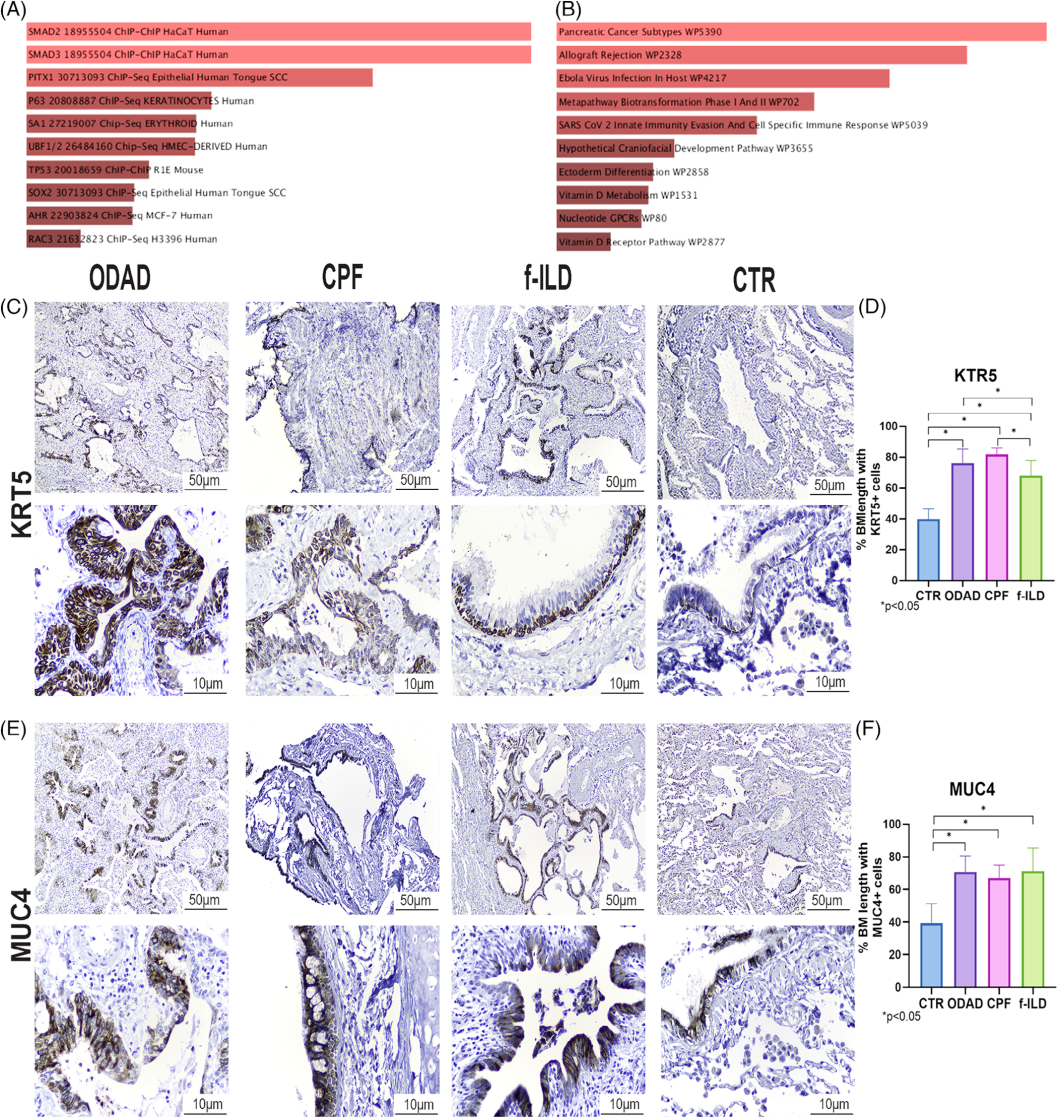
Enrichment analysis and immunohistochemistry of progression‐related genes. The upregulated Progression‐related genes revealed a significant association with the transcription factors SMAD2 and SMAD3: “SMAD2 18955504 ChIP HaCaT Human” *p*‐value = .00002442, *p*_adj_ = .005947; “SMAD3 18955504 ChIP HaCaT Human” *p*_adj_‐value = .00002442, *p*_adj_ = .005947 (A); and “SARS CoV 2 innate immunity evasion and cell specific immune response WP5039” *p*_adj_‐value = .002595, *p*_adj_ = .02180 (B). Immunohistochemical analysis indicated that, in response to injury, KRT5+ basal cell hyperplasia and squamous metaplasia were predominantly found in regions linked to parenchymal remodelling and septal thickening (C). Higher expression of KRT5+ was observed in organizing diffuse alveolar damage (ODAD), coronavirus disease 2019 pulmonary fibrosis (CPF) and fibrosing interstitial lung disease (f‐ILD) compared to the CTR group (*p*_adj_ < .05), and in ODAD and CPF compared to f‐ILD (*p*_adj_ < .05) (D). Goblet cells in areas undergoing parenchymal remodeling exhibited MUC4+ expression (E), with higher expression in ODAD, CPF, and f‐ILD compared to the CTR group (*p*_adj_ < .05) (F). *Note: BM: basement membrane.

To further validate the gene expression findings (Figure S4E–J), we selected two additional markers, KRT5 and MUC4, both widely used in diagnostic IHC. Increased in‐situ expression of the epithelial marker KRT5+ in basal cell hyperplasia was observed in all groups compared to CTR (Figure 4C,D, *p* < .05) consistent with squamous metaplasia reported in COVID‐19 cases.^9^ Similarly, elevated in‐situ MUC4+ expression in bronchial goblet cell hyperplasia (Figure 4E,F) was observed, consistent with findings in idiopathic pulmonary fibrosis (IPF).^10^ The authors addressed MUC4 as part of the TGF‐β1 canonical pathway, suggesting its role in the fibrotic microenvironment.

In conclusion, our study identifies a gene expression profile associated with COVID‐19 progression to pulmonary fibrosis, revealing shared pathways with f‐ILDs. In this way, the KRT5+ cells impair epithelial repair, while MUC4 overexpression exacerbates inflammation and tissue damage, restricting proper regeneration and promoting pulmonary fibrosis. MUC5AC maintains inflammation through mucus hypersecretion, and WNT10a drives fibroblast proliferation and ECM production. Continuous activation of the Wnt pathway by WNT10a contributes to tissue stiffness, which is crucial for sustaining pulmonary fibrosis. Furthermore, we demonstrate increased transcriptomic and in‐situ expression of MUC4 and KRT5, suggesting their pivotal role in fibrosis progression and potential as early diagnostic biomarkers in transbronchial biopsy samples. However, a limitation of our study is that it captures gene expression at a single time‐point, and longitudinal studies are needed to track gene expression changes from the acute infection phase to fibrosis development. Additionally, further validation of these biomarkers in larger cohorts, as well as investigation into their underlying mechanisms, is necessary given their significant potential for clinical practice as immunohistochemical markers for early intervention in fibrotic diseases.

## AUTHOR CONTRIBUTIONS

Conceptualization: Sabrina Setembre Batah and Alexandre Todorovic Fabro; Formal analysis: Sabrina Setembre Batah, Amanda Piveta Schnepper, Robson Francisco Carvalho and João Paulo Facio Almeida; Funding acquisition: Sabrina Setembre Batah and Alexandre Todorovic Fabro; Investigation: Sabrina Setembre Batah, Juliana Rocha Souza Chiappetto, Tales Rubens de Nadai, Marcel Konigkam Santos, Li Siyuan Wada, Danilo Tadao Wada, Andrea Antunes Cetlin, Bruno Guedes Baldi and Suzana Tanni; Methodology: Sabrina Setembre Batah, Andrea Jazel Rodriguez‐Herrera, Maria Júlia Faci do Marco, Juliana Rocha Souza Chiappetto, Mariana Gatto, Simone Alves do Vale, Robson Aparecido Prudente, Amanda Piveta Schnepper and João Paulo Facio Almeida; Project administration: Alexandre Todorovic Fabro; Resources: Robson Francisco Carvalho, José Baddini‐Martinez, Bruno Guedes Baldi, Suzana Tanni and Alexandre Todorovic Fabro; Software: Sabrina Setembre Batah, Amanda Piveta Schnepper, Robson Francisco Carvalho and João Paulo Facio Almeida; Supervision: Vera Luiza Capelozzi, Rosane Duarte Achcar and Alexandre Todorovic Fabro; Validation: Sabrina Setembre Batah, Robson Francisco Carvalho and Alexandre Todorovic Fabro; Visualization: José Baddini‐Martinez, Vera Luiza Capelozzi, Rosane Duarte Achcar and Alexandre Todorovic Fabro; Writing—original draft: Sabrina Setembre Batah and Alexandre Todorovic Fabro; Writing—review & editing: Sabrina Setembre Batah, Robson Francisco Carvalho, José Baddini‐Martinez, Vera Luiza Capelozzi, Suzana Tanni, Rosane Duarte Achcar and Alexandre Todorovic Fabro.

## CONFLICT OF INTEREST STATEMENT

The authors declare no conflict of interest.

## FUNDING INFORMATION

This research was supported by São Paulo Research Foundation (Fapesp 22/02821‐0; 20/13370‐4; 23/10186‐6; 23/04199‐8; 21/09024‐6; 23/10186‐6; 23/04199‐8; 23/10184‐3) and by the National Council for Scientific and Technological Development (CNPq 310415/2021‐7).

## ETHICS STATEMENT

The Research Ethics Committee approved this study, and written informed consent was waived (CAAE: 43040920.0.0000.5440, 03737018.6.0000.5440 and 65315822.0.0000.5411).

## Supporting information



Supporting Information

Supporting Information

Supporting Information

Supporting Information

Supporting Information

## Data Availability

The data that support the findings of this study are available upon request from the corresponding author.
