# Supplementary material for: Transcriptomic profiling reveals the dynamics of fibrotic progression‐related gene expression into post‐coronavirus disease 2019 pulmonary fibrosis
Source: Clin Transl Med. 2024 Nov 13;14(11):e70088. doi: 10.1002/ctm2.70088 (PMC11560857; doi:10.1002/ctm2.70088)
Supplement: Supplementary file 1 — Supporting Information [file CTM2-14-e70088-s004.docx]

**Online Supplementary Material**

**Transcriptomic profiling reveals the dynamics of fibrotic progression-related Gene Expression into Post-COVID-19 Pulmonary Fibrosis**

Sabrina Setembre Batah^1^, Andrea Jazel Rodriguez-Herrera^1^, Maria Júlia Faci do Marco^1^, Juliana Rocha Souza Chiappetto^2^, Mariana Gatto^2^, Simone Alves do Vale^2^, Robson Aparecido Prudente^2^, Amanda Piveta Schnepper^3^, Robson Francisco Carvalho^3^, João Paulo Facio Almeida^4^, Tales Rubens de Nadai^5^, Marcel Konigkam Santos^5^; Li Siyuan Wada^6^, José Baddini-Martinez^7^, Danilo Tadao Wada^8^, Andrea Antunes Cetlin^9^, Vera Luiza Capelozzi^10^, Bruno Guedes Baldi^11^, Suzana Tanni^2^, Rosane Duarte Achcar^12^*, Alexandre Todorovic Fabro^1^

*Co-senior author

**Corresponding authors**

Sabrina Setembre Batah - Department of Pathology and Legal Medicine – FMRP/USP - Avenida Bandeirantes, 3900, Zip code 14049-900 – Ribeirão Preto, São Paulo, Brazil

e-mail: [sabrina.batah@usp.br](mailto:sabrina.batah@usp.br)

Alexandre Todorovic Fabro - Department of Pathology and Legal Medicine – FMRP/USP - Avenida Bandeirantes, 3900, Zip code 14049-900 – Ribeirão Preto, São Paulo, Brazil

e-mail: [fabro@fmrp.usp.br](mailto:fabro@fmrp.usp.br)

**METHODS**

**Study design and patient selection**

Our retrospective study involved data from patients categorized into four groups based on their diagnosis:

**1) Organizing diffuse alveolar damage (ODAD), Sub-acute:** Five samples of COVID-19 autopsies, previously defined as fibrotic phenotype by Batah et al^1^, were obtained using a modified minimally invasive autopsy (MIA)^2^ procedure within one hour of death. The specimens were promptly stored at -80°C, following the protocol described by our research group^1^. The samples were collected during autopsy routine from May/2020 to July/2020 at Hospital das Clínicas, Ribeirão Preto Medical School, University of São Paulo (HCFMRP/USP);

**2)** **COVID-19 pulmonary fibrosis (CPF), Chronic:** Eight transbronchial biopsies were systematically collected and immediately stored in frozen liquid nitrogen, following the established protocol of our research group^3^. The samples were collected during hospital routine from August/2021 to March/2022 at Hospital das Clínicas, Botucatu Medical School, São Paulo State University (FMB/UNESP);

**3) Fibrosing interstitial lung disease (f-ILD), Chronic:** Sixteen formalin-fixed paraffin-embedded(FFPE) samples from surgical lung biopsies were selected from the HCFMRP/USP archive with alternative diagnosis to usual interstitial pneumonia(UIP) pattern by ATS/ERS criteria guidelines^4^;

**4)** **Non-fibrotic lung-Control (CTR):** Six non-neoplastic tissue samples from lung cancer lobectomy were collected and stored immediately in frozen liquid nitrogen. The samples were collected during hospital routine at HCFMRP/USP.

Despite the different methods of lung tissue sampling, the assessment consistently targeted comparable regions—specifically, the bronchovascular axis and the adjacent septal component, both located in the right lower lobe.

Demographic, clinical, radiological and pathological data of all patients enrolled in this study were collected from electronic medical record. This study was approved by the Research Ethics Committee and written informed consent was waived (CAAE: 43040920.0.0000.5440, 03737018.6.0000.5440 and 65315822.0.0000.5411).

**Sample preparation for RNA-Seq**

RNA extraction from frozen and FFPE samples were performed with Purelink RNA Mini kit and MagMAX FFPE DNA/RNA Ultra kit(ThermoFisher Scientific) respectively, according to protocol guidelines. The construction of the cDNA library was carried out with Collibri Stranded RNA Library Prep Kit(ThermoFisher Scientific), following the manufacturer's instructions. Library quantification by qPCR with the Collibri Library Quantification kit(ThermoFisher Scientific) was performed. After normalization, the cDNA library was added together with 1% PhiX and loaded into the cartridge at HiSeq 3000/4000 Illumina for RNA sequencing.

**RNA-Seq analysis**

Initially, the quality of the FASTQ files was assessed using the FASTQC tool. Subsequently, the reads were processed through the nf-core RNA-seq pipeline(https://nf-co.re/rnaseq/3.8.1). Mapping was performed using STAR(https://github.com/alexdobin/STAR), utilizing the GRCh38.p14 genome version from the Gencode(https://www.gencodegenes.org/human/), and quantification was carried out using Salmon(<https://combine-lab.github.io/salmon/>). Differential gene expression (DGE) analysis was performed with DESeq2 program(v1.22.2) in R(v4.2.1). A gene was classified as a significant differential expression gene by adhering to the criteria of an adjusted p-value of ≤ 0.05. All graphical analyses were conducted using R, including principal component analysis (PCA), Enhanced Volcano (v1.18.0), pheatmap (v1.0.12) for hierarchical clustering with Pearson correlation, VennDiagram (v1.7.3), and waterfall plots for the top 20 genes differentially expressed based on log2 Fold Change. The WP Lung Fibrosis gene set (Gene Set Enrichment Analysis - GSEA, systematic name M39477) and Enrichr^5-7^ were employed.

**Histochemistry and Immunohistochemistry**

Immunohistochemical staining (IHC) was performed on 3-μm thick FFPE sections for all samples from each group. Four antibodies were used: MUC4, commercially available monoclonal IgG antibody (BioSB, clone 8G7) with antigen retrieval with Citrate at 1:100 dilution; Cytokeratin 5/6 (KRT5), commercially available monoclonal IgG1 antibody (Dako Omnis, Agilent, clone D5/16 B4) with antigen retrieval with Tris-EDTA, ready-to-use; MUC5AC, commercially available monoclonal IgG antibody (BioSB, clone CLH2) with antigen retrieval with Citrate at 1:50 dilution; and WNT10a commercially available polyclonal IgG antibody (Abcam, ab106522) with antigen retrieval with Citrate at 1:25 dilution. Additionally, Picrosirius Red staining was performed on 3-μm thick FFPE sections for all samples from each group.

The images were captured with a digital camera on microscope (Novel L3000 LED) and analyzed using Image Pro Plus 7 software. Morphometric analysis was performed following the concepts from morphometric standards established by American Thoracic Society and European Thoracic Society (ATS/ERS)^8^. For IHC, ten images of bronchiolar axes were randomly selected at 400x magnification for analysis. The stained area of the bronchiolar epithelial cell basement membrane and the total basement membrane length were measured, and the ratio between these two values was calculated as the percentage of basement membrane length with positive staining. For Picrosirius Red staining, the amount of collagen fibers in the lung parenchyma was measured in ten randomly selected microscopic fields at 200x magnification. The collagen fiber threshold was standardized across all slides after polarization to clearly identify thick collagen fibers in yellow/red (possibly representing collagen type I) and reticular fibers in green (possibly representing collagen type III).

**Statistical analysis**

Statistical analysis was performed with SPSS v.13.0 0 software(SPSS, Inc., Chicago, IL, 2004). The data was evaluated using unpaired T test. Data are expressed as mean ± standard deviation and the p value less than 0.05 was considered statistically significant.

**SUPPLEMENTARY TABLES**

**Table S1 – Demographical data.**

| **DEMOGRAPHIC DATA** | **ODAD** | **CPF** | **f-ILD** | **CTR** |
| --- | --- | --- | --- | --- |
| **Gender (M;F)** | 1;4 | 6;2 | 8;8 | 4;2 |
| **Age (yrs)*** | 70.6 ± 7.1 | 62.1 ± 12.1 | 58.3 ± 10.6 | 60.2 ± 12.8 |
| **Height (cm)*** | 170 ± 0.1 | 165 ± 8.6 | 160 ± 0.1 | 170 ± 6.7 |
| **Weight (kg)*** | 82.7 ± 11.2 | 76.5 ± 12.2 | 81.8 ± 14.2 | 76 ± 14.4 |
| **Body mass index (Kg/m²)*** | 28.8 ± 5.9 | 28.6 ± 5.4 | 30.7 ± 4.8 | 24.8 ± 7.5 |

Note: ODAD: organizing diffuse alveolar damage; CPF: COVID-19 pulmonary fibrosis; f-ILD: Fibrosing interstitial lung disease; CTR: Non-fibrotic lung-Control. *mean ± standard deviation.

**Table S2 – Top 20 differentially expressed genes in ODAD vs CPF.**

|  |  |  |  |  |
| --- | --- | --- | --- | --- |
| **TOP 20 MOST REGULATED GENES** | | |  |  |
|  | **Gene** | **log2FoldChange** | **pvalue** | **padj** |
|  | CDKN1A | 3,38688379 | 1,79389372735024E-22 | 4,97105890786025E-18 |
|  | CELA2B | 7,964510526 | 5,10832791280916E-15 | 9,43712498612364E-12 |
|  | CLDN18 | -5,232982522 | 2,55960877973371E-15 | 5,06637992108577E-12 |
|  | CLIC4 | 3,84927705 | 9,62680071677561E-17 | 2,66768274662569E-13 |
|  | COL1A1 | 2,817713984 | 3,69044700677266E-16 | 9,2969070004252E-13 |
|  | COLGALT1 | 1,719863607 | 6,42659462501737E-17 | 2,43344073967152E-13 |
|  | FAM184A | -2,524484106 | 1,47263095627894E-14 | 2,14779349628662E-11 |
|  | FBLIM1 | 2,097286719 | 5,88803058961823E-16 | 1,35969346390759E-12 |
|  | FKBP10 | 2,431989187 | 3,78429038769819E-17 | 1,74777451555841E-13 |
|  | GALNT5 | -3,818196454 | 6,62935142292007E-15 | 1,14816223300336E-11 |
|  | HS3ST3A1 | 2,930204645 | 1,07722926208155E-13 | 1,24379583673091E-10 |
|  | IL4R | 5,776657118 | 1,18444214084587E-14 | 1,86406080626298E-11 |
|  | NOTCH4 | -7,876590673 | 2,96084158443276E-14 | 3,72944914300983E-11 |
|  | RCN3 | 3,073807273 | 3,62709835897674E-20 | 5,02552613128023E-16 |
|  | RNA5-8SN1 | -23,64020499 | 2,5493435417139E-14 | 3,36404089925875E-11 |
|  | RNU6-4P | 25,4123095 | 8,89877832363761E-17 | 2,66768274662569E-13 |
|  | SERPINE1 | 4,25542799 | 1,21082221907306E-14 | 1,86406080626298E-11 |
|  | SOD2 | 2,914689616 | 3,94799615372987E-19 | 2,87074551704487E-15 |
|  | THBS2 | 3,457919746 | 7,02519790602006E-17 | 2,43344073967152E-13 |
|  | TOM1 | 1,340566504 | 4,1438353246651E-19 | 2,87074551704487E-15 |

Note: ODAD: organizing diffuse alveolar damage; CPF: COVID-19 pulmonary fibrosis.

**Table S3 – Expression of WP Lung Fibrosis Gene Set in ODAD vs CPF**

| **WP LUNG FIBROSIS GENE SET** | | |  |  |
| --- | --- | --- | --- | --- |
|  | **Gene** | **log2FoldChange** | **pvalue** | **padj** |
|  | ATP11A | -0.376184686278173 | 0.371842457088408 | 0.602193111353916 |
|  | BMP7 | 0.488199472839765 | 0.500904626687426 | 0.709459141841823 |
|  | CALCA | -3.08708898970383 | NA | NA |
|  | CCL11 | 3.17154156253219 | 0.00715082977768525 | 0.0483255483064977 |
|  | CCL2 | -0.537804582006968 | 0.314036448302599 | 0.54641868761229 |
|  | CCL3 | -1.01156923943782 | 0.703170150707426 | NA |
|  | CCL4 | -0.53322540738041 | 0.684527911046778 | 0.83814337962715 |
|  | CCL5 | 2.59388167718932 | 2.67717372304561e-05 | 0.000803761224694657 |
|  | CCN2 | -0.263275216528847 | 0.570016822078809 | 0.760177879427589 |
|  | CCR2 | 2.43051480270204 | 0.00107097376725748 | 0.0126260199291973 |
|  | CCR3 | 1.1280219369087 | 0.415143147985014 | 0.640286735337714 |
|  | CEBPB | -1.48971968524292 | 5.61855904048223e-05 | 0.00140646693379226 |
|  | CMA1 | 0.597515156728072 | 0.805139542310785 | NA |
|  | CSF2 | 2.24823064314099 | 0.0705836680931961 | 0.220377866446466 |
|  | CSF3 | -4.4380789361532 | NA | NA |
|  | CXCL2 | -2.8887309356972 | NA | NA |
|  | CXCL8 | -2.45548548220303 | 0.00754080700575729 | 0.0500510905237222 |
|  | CYSLTR2 | 1.52685558910836 | 0.112554627898977 | 0.295752066537886 |
|  | DPP9 | -0.777235685051606 | 0.00529285445115326 | 0.0392271435399594 |
|  | DSP | 1.040293878127 | 0.0881196910717321 | 0.254207199251798 |
|  | EDN1 | 1.41323788782367 | 0.0126608298144649 | 0.071411409523435 |
|  | EGF | 0.909531674925654 | 0.299345700876259 | 0.531755590449086 |
|  | ELMOD2 | -0.36662817384395 | 0.438682046005691 | 0.660095470072963 |
|  | ELN | 0.287157984887537 | 0.545878955514286 | 0.742649197941032 |
|  | FAM13A | 0.248425953621009 | 0.543773449585398 | 0.741596833577487 |
|  | FGF1 | 3.02411052042532 | 0.00215097221632541 | 0.0208630000303092 |
|  | FGF2 | -3.32570029119256 | 0.0152282881026258 | 0.080841205289629 |
|  | FGF7 | -1.67715179097653 | 0.00309747389148641 | 0.0268735438343707 |
|  | GREM1 | -3.21278912297076 | 0.0234684395447653 | 0.108100719452292 |
|  | HGF | -1.25094597210206 | 0.00972305998468765 | 0.0594912155521483 |
|  | HMOX1 | -2.02651121035484 | 4.06308181912037e-07 | 3.16269832274283e-05 |
|  | IGF1 | -1.77614545399058 | 0.00825887292746809 | 0.0532246683900969 |
|  | IL12B | -1.14886444401391 | 0.485419993965392 | NA |
|  | IL13 | -1.99586117683626 | 0.324657455704801 | NA |
|  | IL1B | -1.24165532649478 | 0.224988744106371 | 0.447686418494798 |
|  | IL4 | 0.601559681181928 | 0.705393663568149 | NA |
|  | IL4 | 0.601559681181928 | 0.705393663568149 | NA |
|  | IL5 | 2.48603363290902 | 0.0634324657412989 | 0.205973407330341 |
|  | IL6 | -3.88309619969333 | 9.34114286664489e-07 | 6.34442181317639e-05 |
|  | MECP2 | -0.43632005524113 | 0.083352327073619 | 0.245538039283199 |
|  | MMP2 | -1.98387030726248 | 5.07823012520247e-05 | 0.00130307086666907 |
|  | MMP9 | -2.51577266413602 | 0.000578140985195701 | 0.00805771035104222 |
|  | MT2A | -2.94342215727304 | 1.90991804719102e-08 | 2.68463048785428e-06 |
|  | MUC5B | 3.08503421748972 | 0.00341348102606488 | 0.0289269029704232 |
|  | NFE2L2 | 0.424000372752593 | 0.148338109554383 | 0.348909683405145 |
|  | PARN | 0.781626263469446 | 0.076762055517451 | 0.232806536110768 |
|  | PDGFA | -0.346532905867103 | 0.328584067192044 | 0.561340055368643 |
|  | PDGFB | -0.554014220312019 | 0.491382832121036 | 0.702109397798599 |
|  | PLAU | 0.4963661497815 | 0.153672044193757 | 0.35638179066476 |
|  | PTX3 | -5.51866781045033 | 4.65088189455103e-12 | 2.3866775588871e-09 |
|  | RTEL1 | -0.90184504078285 | 0.00291210341729947 | 0.0256344656279497 |
|  | SERPINA1 | 0.405483897970538 | 0.695804612403051 | 0.845677263785129 |
|  | SFTPA1 | 1.12323790743569 | 0.116352327536095 | 0.302093071147074 |
|  | SFTPA2 | 1.7263854305513 | 0.0197379978855836 | 0.096147731748675 |
|  | SFTPC | 3.86667132754474 | 1.90228703044012e-05 | 0.000617263183846911 |
|  | SKIL | 0.0332602463363541 | 0.931428498643754 | 0.970586527481907 |
|  | SMAD7 | -0.274227917715184 | 0.547817837411831 | 0.743615338131008 |
|  | SPP1 | -3.14230817350947 | 1.57560952799477e-06 | 9.29816563599067e-05 |
|  | STN1 | 1.07364380583739 | 0.000549344552265609 | 0.00775096073718548 |
|  | TERT | -3.76732888289388 | 0.000646773695067772 | 0.00872577695424685 |
|  | TGFA | -1.76604025443497 | 0.133202529143759 | 0.328310164342198 |
|  | TGFB1 | -0.457164217970363 | 0.190144658902846 | 0.40500373888215 |
|  | TIMP1 | -2.3882236012844 | 1.90159759284422e-10 | 5.43249184487692e-08 |
|  | TNF | -0.963774471552216 | 0.760526696368591 | NA |

Note: ODAD: organizing diffuse alveolar damage; CPF: COVID-19 pulmonary fibrosis.

**Table S4 – Top 20 differentially expressed genes in CPF vs f-ILD.**

| **TOP 20 MOST REGULATED GENES** | | |  |  |
| --- | --- | --- | --- | --- |
|  | **Gene** | **log2FoldChange** | **pvalue** | **padj** |
|  | AGR2 | -2,401511501 | 2,19100698796774E-05 | 0,0254241964416623 |
|  | AZGP1 | -3,462421506 | 2,9707709807886E-06 | 0,00731106140760186 |
|  | CDH23 | 0,914849964835127 | 2,23483933941061E-05 | 0,0254241964416623 |
|  | CLCA2 | -4,175563405 | 2,27630835759927E-05 | 0,0254241964416623 |
|  | COL1A1 | 2,101772736 | 2,19392286493748E-05 | 0,0254241964416623 |
|  | DXO | -22,00414909 | 5,41827585984421E-16 | 4,23618867642153E-12 |
|  | FOSB | 2,668499353 | 1,20148314842682E-05 | 0,0187871914975674 |
|  | GNL3 | -1,389542014 | 2,21093970009374E-05 | 0,0254241964416623 |
|  | GOLGA8M | -5,268151832 | 6,39390331622904E-06 | 0,0123943664374371 |
|  | GSTA1 | -3,66216867 | 3,11790273200923E-05 | 0,0325119106187049 |
|  | HSPA1A | -19,93802988 | 2,35279896602282E-10 | 9,19748329134419E-07 |
|  | LYPD6 | 2,447461038 | 3,54586925471857E-05 | 0,0331548479340306 |
|  | MOSPD3 | 1,37433143 | 2,1075007559732E-05 | 0,0254241964416623 |
|  | PPP1R10 | -8,672940166 | 3,11705879667528E-06 | 0,00731106140760186 |
|  | RMRP | -30 | 1,09577981633356E-27 | 2,57015155921036E-23 |
|  | RNU6-3P | -21,67615846 | 5,48472947979229E-12 | 2,57288659897056E-08 |
|  | RNU6-6P | -21,75295287 | 4,61541344319462E-12 | 2,57288659897056E-08 |
|  | SLPI | -2,874362223 | 3,50564030793179E-06 | 0,00747498122023092 |
|  | SNORA57 | -26,95944454 | 9,97756843153834E-18 | 1,17011933780866E-13 |
|  | TREX2 | 1,467816558 | 3,18812169784785E-05 | 0,0325119106187049 |

Note: CPF: COVID-19 pulmonary fibrosis; f-ILD: Fibrosing interstitial lung disease.

**Table S5 – Top 20 differentially expressed genes in ODAD vs f-ILD.**

| **TOP 20 MOST REGULATED GENES** | | |  |  |
| --- | --- | --- | --- | --- |
|  | **Gene** | **log2FoldChange** | **pvalue** | **padj** |
|  | BAG6 | -1,132216548 | 0,390963006681205 | 0,994798902604771 |
|  | CLIC1 | -18,78927774 | 8,67928567918027E-08 | 0,000179383476417298 |
|  | DBH | 2,66842995 | 3,82942790703219E-05 | 0,0526182392216533 |
|  | DDX39B | -3,503361651 | 1,8130374870753E-10 | 8,29937364668052E-07 |
|  | ENTREP3 | -2,472365574 | 4,0734073328162E-05 | 0,0526182392216533 |
|  | GLRX5 | 3,066263297 | 7,21644530139473E-06 | 0,012644951595248 |
|  | HLA-F | -6,159077204 | 2,54593413578374E-05 | 0,0375852619416988 |
|  | MIR663A | -23,81600866 | 5,15642363196999E-22 | 1,06572963625556E-17 |
|  | PPP1R10 | -22,36441887 | 2,37353651478064E-16 | 1,63520842291621E-12 |
|  | RMRP | -30 | 1,20938604307926E-17 | 1,24977953691811E-13 |
|  | RN7SL688P | -5,425387759 | 7,75788904101401E-05 | 0,0641360202798711 |
|  | RNA5SP42 | 3,927460369 | 6,22734790160708E-05 | 0,0636485090966946 |
|  | RNA5SP532 | 5,098378135 | 7,3417562968345E-06 | 0,012644951595248 |
|  | RNU6-6P | -18,93976911 | 6,83759375134121E-08 | 0,000157021541836356 |
|  | RNU6-9 | -21,35054032 | 1,17268728059329E-09 | 4,03951678588369E-06 |
|  | RXRB | -8,595428975 | 1,52966690019498E-09 | 4,51645078474713E-06 |
|  | SESN2 | -7,775797757 | 2,09852163750305E-05 | 0,0333632655414716 |
|  | SSTR3 | 2,978633303 | 7,67237171070071E-05 | 0,0641360202798711 |
|  | U2AF1 | -1,80030122 | 6,467092563531E-05 | 0,0636485090966946 |
|  | VASN | -22,31548681 | 2,00778344461983E-10 | 8,29937364668052E-07 |

Note: ODAD: organizing diffuse alveolar damage; f-ILD: Fibrosing interstitial lung disease.

**Table S6 – Expression of WP Lung Fibrosis Gene Set in CPF vs f-ILD.**

| **WP LUNG FIBROSIS GENE SET** | | |  |  |
| --- | --- | --- | --- | --- |
|  | **Gene** | **log2FoldChange** | **pvalue** | **padj** |
|  | ATP11A | 0,175882691897868 | 0,521349988876313 | 0,799242771978982 |
|  | BMP7 | 0,163082760047131 | 0,633598467808472 | 0,859778128447032 |
|  | CCL11 | 0,48565980087172 | 0,348604735733574 | 0,679463463485903 |
|  | CCL2 | 0,288251663300511 | 0,510344643511039 | 0,792304316491357 |
|  | CCL3 | -0,283267875195249 | 0,783288737983632 | 0,925146136899275 |
|  | CCL4 | 0,584122274641907 | 0,508126056670511 | 0,790935711795456 |
|  | CCL5 | -1,618915604 | 0,374832616731661 | NA |
|  | CCN2 | 0,504396102728435 | 0,119414785992907 | 0,436613219869623 |
|  | CCR2 | 0,144985470074974 | 0,742646461696059 | 0,90940653435737 |
|  | CCR3 | 0,145674272095338 | 0,862441387468801 | NA |
|  | CEBPB | 0,239191255937661 | 0,370180213122431 | 0,694707640897349 |
|  | CMA1 | 1,847427532 | 0,0309222289608657 | NA |
|  | CSF2 | 0,0177794438496915 | 0,981959570460706 | NA |
|  | CSF3 | 0,0329185553659635 | 0,955512643631228 | 0,98545152397941 |
|  | CXCL2 | 0,903563835556769 | 0,0358157133420477 | 0,262219982102811 |
|  | CXCL8 | -1,374528179 | 0,0425596714611163 | 0,283685048106996 |
|  | CYSLTR2 | -0,318450543315457 | 0,494044019408068 | 0,781838757091754 |
|  | DSP | -1,41169247 | 0,00115678083349399 | 0,0907434596976636 |
|  | EDN1 | -0,68157447004628 | 0,0787322626293657 | 0,368595852289775 |
|  | EGF | -0,182384805655062 | 0,729533571252479 | 0,903765662669601 |
|  | ELMOD2 | 0,486739704197677 | 0,229887132101909 | 0,570823913132572 |
|  | ELN | 0,505362002591468 | 0,118726515448127 | 0,435795057877281 |
|  | FAM13A | -0,410367177908295 | 0,114488357280049 | 0,430340451923644 |
|  | FGF1 | -0,703383288720769 | 0,109514015384945 | 0,421632077386091 |
|  | FGF7 | -0,270588092240487 | 0,563436133049302 | 0,823850546262037 |
|  | GREM1 | 1,923774976 | 0,0652565881127288 | 0,338477061960207 |
|  | HGF | -0,0888789691899032 | 0,795057794934528 | 0,9309610393984 |
|  | HMOX1 | 0,536052691191911 | 0,0542237549058435 | 0,310076041912756 |
|  | IGF1 | -0,264638623711938 | 0,615543348298548 | 0,85085782879573 |
|  | IL12B | -0,761130774972721 | 0,436156462745655 | NA |
|  | IL13 | 0,982695252733665 | 0,314613540834703 | NA |
|  | IL1B | 0,00647120170984925 | 0,990538308558693 | 0,997387669552813 |
|  | IL4 | -0,231149217594057 | 0,777680809327303 | NA |
|  | IL5 | 0,127218655099517 | 0,866720575352426 | NA |
|  | IL6 | -0,0249496301747481 | 0,969609134888964 | 0,989564975146665 |
|  | MMP2 | 0,721919268969797 | 0,0345427436008512 | 0,258025493999352 |
|  | MMP9 | 0,712767375774236 | 0,177078832052441 | 0,512294567944116 |
|  | MT2A | 0,20973602230903 | 0,625966638331759 | 0,856277502312976 |
|  | MUC5B | -1,40377073 | 0,0596876172081185 | 0,324744389147859 |
|  | PARN | -0,611377085811226 | 0,57316171816692 | 0,829897053985073 |
|  | PDGFB | 0,0610166812138154 | 0,866931008708444 | 0,956167384134334 |
|  | PLAU | 0,154721851977329 | 0,594933169458918 | 0,839972938312869 |
|  | PTX3 | -0,427628394107814 | 0,368285275859257 | 0,693602950480077 |
|  | SERPINA1 | 1,31800265 | 0,0152997082525678 | 0,19241536571795 |
|  | SFTPA1 | -0,257534217039168 | 0,606763931826585 | 0,846718706627353 |
|  | SFTPA2 | -0,214936060058745 | 0,619406077489203 | 0,85251362488998 |
|  | SFTPC | -0,126496354627237 | 0,800076381383552 | 0,933525570031797 |
|  | SMAD7 | 0,304264785925601 | 0,296806088729844 | 0,633820018322149 |
|  | SPP1 | -0,316214992133088 | 0,615674470688821 | 0,850901226209787 |
|  | TERT | 1,853860081 | 0,0198471063882639 | 0,20940795336785 |
|  | TGFA | -0,247354647066609 | 0,488190477272521 | 0,778174684752135 |
|  | TGFB1 | 0,954143316389733 | 0,0018239133087505 | 0,102589656251182 |
|  | TIMP1 | 0,399833435684274 | 0,181212892270801 | 0,517451715146291 |
|  | TNF | 3,617458034 | 0,0158551700989602 | 0,194296245909671 |

Note: CPF: COVID-19 pulmonary fibrosis; f-ILD: Fibrosing interstitial lung disease.

**Table S7 – Expression of** **WP Lung Fibrosis Gene Set in ODAD vs f-ILD.**

| **WP LUNG FIBROSIS GENE SET** | | |  |  |
| --- | --- | --- | --- | --- |
|  | **Gene** | **log2FoldChange** | **pvalue** | **padj** |
|  | ATP11A | 0,295991382161279 | 0,256034712778406 | 0,96504641539285 |
|  | BMP7 | 0,512812708354645 | 0,223684473582205 | 0,960505035509051 |
|  | CCL11 | 1,087921951 | 0,154081982028998 | 0,937798362185614 |
|  | CCL2 | -0,437130478320112 | 0,359221421121059 | 0,989448400224139 |
|  | CCL3 | -0,068744562310338 | 0,960897629608721 | 0,998133257253993 |
|  | CCL4 | 1,012220422 | 0,230566201732759 | 0,962478982436802 |
|  | CCL5 | 1,762281197 | 0,427554093627061 | NA |
|  | CCN2 | 0,221081270008387 | 0,565310704869744 | 0,99618466970255 |
|  | CCR2 | 0,0637008574330559 | 0,936618156220576 | 0,998133257253993 |
|  | CCR3 | 0,311169411004537 | 0,706240407316725 | NA |
|  | CEBPB | -0,37823403044975 | 0,414764022055097 | 0,995654855447069 |
|  | CMA1 | 2,0540236 | 0,0394996814665311 | NA |
|  | CSF2 | 1,065251752 | 0,280109936920793 | NA |
|  | CSF3 | -0,431457855935183 | 0,541800552092689 | 0,99618466970255 |
|  | CXCL2 | -0,349961473845493 | 0,600152917513649 | 0,99618466970255 |
|  | CXCL8 | -2,363372372 | 0,0105966495974294 | 0,611580034419681 |
|  | CYSLTR2 | -0,346393873605627 | 0,627858457873224 | 0,99618466970255 |
|  | EDN1 | -0,111223383984151 | 0,809340872850671 | 0,99618466970255 |
|  | EGF | -0,268872189166105 | 0,735050119518581 | NA |
|  | ELMOD2 | 1,195991491 | 0,0209059452596602 | 0,704779537694186 |
|  | ELN | 0,895781480926581 | 0,0307270594501679 | 0,74789959002828 |
|  | FAM13A | -0,398445309399695 | 0,258866094971742 | 0,966721059238432 |
|  | FGF1 | -0,654849231456592 | 0,341390322293397 | 0,987938277955745 |
|  | FGF7 | -0,69797008533571 | 0,261141945609258 | 0,968304734876286 |
|  | GREM1 | 0,162367210938639 | 0,901307675735214 | 0,998133257253993 |
|  | HGF | -0,382170750253748 | 0,365479669109895 | 0,991381898473092 |
|  | HMOX1 | -0,884845416231409 | 0,11013696729907 | 0,897839157456446 |
|  | IGF1 | -0,585713937881566 | 0,220540014416644 | 0,95999678160741 |
|  | IL12B | -2,220001398 | 0,0891273705534068 | NA |
|  | IL13 | -0,058253236802249 | 0,95405529832463 | NA |
|  | IL1B | -0,992927159300558 | 0,152807239031871 | 0,934880809712935 |
|  | IL4 | -0,446816098437756 | 0,743514521321322 | NA |
|  | IL5 | 0,827808674180261 | 0,433294586488957 | NA |
|  | IL6 | -2,300434148 | 0,00720756162450713 | 0,533611828477553 |
|  | MMP2 | 0,141078245173158 | 0,679420145545022 | 0,99618466970255 |
|  | MMP9 | -0,152818132738781 | 0,853215958893087 | 0,997937055502936 |
|  | MT2A | -1,363483953 | 0,0200169458482844 | 0,701716654786554 |
|  | MUC5B | -0,577929402907519 | 0,505148180328996 | 0,99618466970255 |
|  | PARN | -1,651717854 | 0,298526600508724 | 0,977938447118885 |
|  | PDGFA | -0,166774195329956 | 0,660541124137144 | 0,99618466970255 |
|  | PDGFB | -0,0907065275609708 | 0,865045519768411 | 0,998133257253993 |
|  | PLAU | 0,190351990423335 | 0,629296727562582 | 0,99618466970255 |
|  | PTX3 | -3,50782154 | 0,00102767923093813 | 0,274234303502128 |
|  | SERPINA1 | 0,026047060204207 | 0,954739100622768 | 0,998133257253993 |
|  | SFTPA1 | 0,597854554710568 | 0,300213258332955 | 0,977938447118885 |
|  | SFTPA2 | 0,0460291404269325 | 0,938124768571403 | 0,998133257253993 |
|  | SFTPC | 1,142852047 | 0,0831041224039465 | 0,863797540694461 |
|  | SMAD7 | 0,257781463133346 | 0,524709588889988 | 0,99618466970255 |
|  | SPP1 | -0,533211432259713 | 0,389606639627395 | 0,994798902604771 |
|  | TERT | 1,021038937 | 0,220572221615529 | 0,95999678160741 |
|  | TGFA | 0,377980517458568 | 0,333052684552817 | 0,984879725526871 |
|  | TGFB1 | 0,746251908204756 | 0,0282574204539421 | 0,730591284687173 |
|  | TIMP1 | -0,464630069568083 | 0,341466711592242 | 0,987977904698364 |
|  | TNF | -2,656759508 | 0,451173483570954 | NA |

Note: ODAD: organizing diffuse alveolar damage; f-ILD: Fibrosing interstitial lung disease.

**Table S8 – Fibrosis-related genes (86) commonly expressed in CPF and f-ILD.**

| **Gene** | **Log2FoldChange CPF vs. CTR** | **padj CPF vs. CTR** | **Log2FoldChange f-ILD vs. CTR** | **padj**  **f-ILD vs. CTR** |
| --- | --- | --- | --- | --- |
| AADACL2 | 4,4264 | 0,0087 | 3,0985 | 0,022 |
| ABCB4 | 2,2013 | 0,0356 | 1,565 | 0,0277 |
| ABTB3 | 1,4770 | 0,0182 | 1,464 | 0,0005 |
| AGPAT1 | 0,0513 | 0,9973 | 7,0463 | 0,0085 |
| ALDH3B2 | 4,0181 | 0,0306 | 2,082 | 0,0467 |
| AQP5 | 2,4373 | 0,0399 | 1,4379 | 0,0038 |
| ATP12A | 4,6769 | 0,0076 | 2,8971 | 0,0029 |
| BARX2 | 4,5331 | 0,0045 | 3,7321 | 0,0009 |
| BCL2L15 | 3,3450 | 0,0137 | 2,3363 | 0,0001 |
| BEST4 | 3,3316 | 0,0224 | 1,5976 | 0,0416 |
| BPIFB1 | 4,8616 | 0,0013 | 2,0278 | 0,0146 |
| BRD2 | 0,8308 | 2,87E-08 | 7,2259 | 0,0225 |
| BRPF3 | 0,5318 | 0,0123 | 0,7184 | 5,67E-08 |
| CD27 | 1,4868 | 0,0366 | 1,6851 | 0,0001 |
| CD38 | 1,7560 | 0,005 | 2,1661 | 2,08E-09 |
| CDON | 1,3668 | 0,021 | 1,1355 | 0,0004 |
| CKMT1B | 4,3702 | 0,0323 | 3,052 | 0,0027 |
| CLCA2 | 7,1447 | 0,0001 | 3,3993 | 0,0068 |
| CLDN3 | 2,1072 | 0,0194 | 1,5044 | 0,0028 |
| CLIC1 | 11,1979 | 0,0007 | 23,4575 | 9,47E-13 |
| COL19A1 | 3,1484 | 0,01 | 2,6196 | 0,0014 |
| CSNK2B | 6,3193 | 0,0303 | 3,8498 | 0,017 |
| DEFA1 | 5,6856 | 0,0104 | 3,7474 | 0,0016 |
| DMRT3 | 5,5546 | 0,0465 | 3,7518 | 0,0002 |
| DSC3 | 5,7172 | 0,0001 | 2,7717 | 0,0049 |
| ERN2 | 4,0167 | 0,0064 | 2,5726 | 0,0003 |
| FAM83B | 3,6807 | 0,0085 | 2,871 | 0,0103 |
| FAT2 | 4,0155 | 0,0004 | 3,2416 | 1,01E-07 |
| FOXA3 | 3,4351 | 0,0452 | 1,9441 | 0,0129 |
| GABRP | 4,8980 | 0,0015 | 1,7022 | 0,0435 |
| GJB4 | 5,9461 | 0,008 | 2,6275 | 0,0392 |
| HSFX2 | 7,1465 | 0,0003 | 4,8884 | 0,0012 |
| IER3 | 20,3934 | 4,37E-07 | 7,7182 | 0,0112 |
| IGHG1 | 2,8071 | 0,0469 | 2,9794 | 0,0002 |
| IGHG2 | 3,0998 | 0,0204 | 3,9871 | 2,12E-09 |
| IGHG3 | 3,3381 | 0,0007 | 3,3107 | 8,81E-08 |
| IGKC | 2,9879 | 0,0062 | 2,1751 | 0,0011 |
| IGLC2 | 3,2086 | 0,005 | 2,1458 | 0,0053 |
| IGLV1-44 | 3,6520 | 0,0013 | 2,0921 | 0,0069 |
| IGLV1-47 | 3,0381 | 0,0103 | 2,517 | 0,0003 |
| IGLV1-51 | 3,4756 | 0,0003 | 1,7932 | 0,0273 |
| IGLV2-8 | 2,6923 | 0,0067 | 2,2482 | 0,0039 |
| IGLV6-57 | 3,6453 | 0,0087 | 2,2128 | 0,0075 |
| JCHAIN | 2,2188 | 0,0004 | 2,4126 | 0,0008 |
| KCNA1 | 5,8639 | 0,0121 | 2,9461 | 0,0078 |
| LGALS9C | 6,4550 | 0,0096 | 4,2096 | 0,0127 |
| LST1 | 22,8485 | 6,88E-09 | 2,2673 | 0,0252 |
| MUC2 | 6,7963 | 0,0103 | 5,9019 | 0,0002 |
| MUC5AC | 6,6669 | 2,91E-05 | 8,2283 | 0,0001 |
| PART1 | 3,4009 | 0,0413 | 2,6068 | 0,0005 |
| PAX9 | 3,4488 | 0,0269 | 2,3215 | 0,0011 |
| POU2AF1 | 3,9046 | 0,0003 | 4,4262 | 3,11E-21 |
| PRH1 | 6,2002 | 0,0006 | 2,1466 | 0,0461 |
| PSCA | 3,9183 | 0,0072 | 3,0788 | 0,0025 |
| PTPRZ1 | 4,4949 | 0,0028 | 1,9915 | 0,0055 |
| RPS18 | 6,7249 | 0,0234 | 3,8256 | 0,0377 |
| SIX2 | 4,0227 | 0,0111 | 3,9081 | 0,0001 |
| SIX4 | 3,0927 | 0,0145 | 1,4743 | 0,008 |
| SLC39A7 | -10,6510 | 0,019 | 8,7428 | 2,43E-05 |
| SLPI | 2,6106 | 0,0038 | -1,3602 | 0,0001 |
| SOX21 | 4,1753 | 0,0032 | 3,3752 | 0,0004 |
| TFF3 | 4,7985 | 0,0013 | 1,3324 | 0,05 |
| TGFBR3L | 2,8480 | 0,0092 | 2,5478 | 0,0002 |
| TPSD1 | 3,6081 | 0,0246 | 3,9518 | 5,50E-08 |
| TRIM29 | 3,2534 | 0,033 | 1,9144 | 0,0013 |
| VSIG1 | 4,1339 | 0,027 | 3,3719 | 0,0012 |
| WFDC2 | 2,5250 | 0,0367 | 2,3125 | 2,85E-07 |
| WNT10A | 3,2228 | 0,0235 | 3,5804 | 1,52E-07 |
| WNT5B | 1,5967 | 0,0447 | 1,6143 | 1,9155E-05 |
| WSCD2 | 3,4202 | 0,0086 | 1,8373 | 0,0002 |
| ZNF648 | 4,4700 | 0,0421 | 4,7243 | 0,0005 |
| ANKRD1 | -2,4140 | 0,001 | -4,5646 | 9,0329E-07 |
| CXCL3 | -2,8708 | 0,0159 | -1,8675 | 0,0032 |
| CYP3A5 | -1,8314 | 0,0023 | -1,3231 | 0,0347 |
| DUSP1 | -1,5623 | 0,0013 | -1,2515 | 0,0102 |
| GADD45B | -1,4823 | 1,22E-06 | -1,2238 | 0,0009 |
| HMGN2 | -0,7144 | 0,0282 | -1,1094 | 1,62E-12 |
| HSD17B14 | -1,2778 | 0,0437 | -1,436 | 0,0001 |
| JUN | -1,9645 | 3,37E-12 | -1,4177 | 0,0088 |
| KMO | -2,7159 | 1,99E-05 | -2,3879 | 0,0007 |
| LOC101928093 | -3,6958 | 0,0093 | -3,2577 | 0,0004 |
| OLR1 | -2,8528 | 0,0162 | -2,4952 | 0,0004 |
| RGS6 | -2,3221 | 0,0021 | -1,5505 | 0,0001 |
| RPL22 | -0,8144 | 0,0072 | -1,6776 | 8,62E-06 |
| RSRC1 | -0,7758 | 0,0339 | -1,12 | 0,0011 |
| SNORA38B | -2,5258 | 0,006 | -3,2957 | 3,94E-09 |

Note: CPF: COVID-19 pulmonary fibrosis; f-ILD: Fibrosing interstitial lung disease.

**Table S9 – Progression-related genes (31) commonly expressed in ODAD, CPF and f-ILD.**

| **Gene** | **Log2FoldChange ODAD vs. CTR** | | **padj ODAD vs. CTR** | **Log2FoldChange CPF vs. CTR** | **padj CPF vs. CTR** | **Log2FoldChange f-ILD vs. CTR** | **padj f-ILD vs. CTR** |
| --- | --- | --- | --- | --- | --- | --- | --- |
| C4B | | 2,3449 | 1,64E-05 | 9,3989 | 7,63E-09 | 1,243 | 0,0232 |
| CDC20B | | 3,7480 | 0,0462 | 4,4297 | 0,0085 | 3,9176 | 0,0001 |
| CHST6 | | 2,4400 | 0,0118 | 3,4732 | 0,0071 | 2,0586 | 0,0002 |
| COL17A1 | | 2,8493 | 0,0389 | 2,5707 | 0,0239 | 2,7746 | 7,01E-11 |
| CXCL6 | | 5,7556 | 0,0105 | 6,6307 | 0,0009 | 4,4209 | 0,0011 |
| CYP24A1 | | 6,4235 | 1,22E-06 | 6,5782 | 0,0005 | 3,3614 | 0,0006 |
| EYA2 | | 2,4012 | 0,0332 | 2,9133 | 0,0453 | 1,885 | 0,0009 |
| FAM30A | | 4,5786 | 0,0022 | 4,0593 | 0,0007 | 4,4121 | 1,93E-09 |
| HAPLN3 | | 2,4022 | 0,0061 | 1,2658 | 0,0125 | 1,6745 | 5,72E-06 |
| HLA-A | | 3,2571 | 0,0058 | 3,9756 | 0,0491 | 2,7566 | 0,0005 |
| HLA-DQB1 | | 5,8852 | 0,0004 | 6,9366 | 0,0001 | 2,7335 | 0,0135 |
| HS6ST1 | | 1,2628 | 0,0417 | 1,2777 | 0,0369 | 0,8868 | 0,0015 |
| KRT15 | | 3,4183 | 0,0092 | 5,7699 | 0,0002 | 2,0593 | 0,0025 |
| KRT5 | | 4,1550 | 0,0212 | 5,8125 | 2,32E-05 | 2,5698 | 0,0068 |
| KRT6A | | 8,1578 | 1,58E-09 | 7,3459 | 0,0013 | 3,312 | 0,0167 |
| MIR205HG | | 4,0054 | 0,0077 | 3,6205 | 0,0384 | 3,0664 | 1,03E-05 |
| MUC16 | | 2,8942 | 0,0272 | 6,926 | 1,94E-08 | 3,74 | 3,22E-06 |
| MUC4 | | 5,1317 | 0,0021 | 4,8346 | 0,003 | 4,3279 | 0,0033 |
| P2RY2 | | 1,2828 | 0,0335 | 1,3231 | 0,0092 | 0,8937 | 0,0277 |
| PITX1 | | 3,7811 | 0,0002 | 4,3394 | 0,003 | 2,7316 | 0,0009 |
| S100A2 | | 4,6088 | 0,0007 | 3,8058 | 0,0079 | 3,2904 | 0,0001 |
| TFAP2A | | 3,8983 | 0,0044 | 3,8399 | 0,0027 | 4,563 | 1,08E-10 |
| TP53AIP1 | | 3,6809 | 0,0430 | 5,1396 | 0,0013 | 2,2411 | 0,0398 |
| DDO | | -2,0968 | 0,0463 | -1,4494 | 0,0454 | -2,2294 | 7,21E-06 |
| GARIN4 | | -3,7266 | 0,0279 | -6,3655 | 1,67E-05 | -4,181 | 0,0003 |
| INHBA | | -2,6311 | 4,44E-05 | -2,7825 | 4,93E-06 | -1,9903 | 1,33E-06 |
| KRT79 | | -4,6342 | 0,0266 | -4,2107 | 0,0384 | -2,2346 | 0,0378 |
| LOC124902436 | | -4,7854 | 0,0074 | -3,5966 | 0,0054 | -3,4654 | 3,13E-05 |
| LPL | | -2,6528 | 0,0006 | -1,8093 | 0,0471 | -1,3272 | 0,009 |
| LTK | | -2,0998 | 0,0171 | -1,3516 | 0,028 | -1,5183 | 0,0054 |
| MME | | -2,5429 | 0,0084 | -2,7276 | 0,0039 | -2,7274 | 2,89E-06 |

Note: ODAD: organizing diffuse alveolar damage; CPF: COVID-19 pulmonary fibrosis; f-ILD: Fibrosing interstitial lung disease.

**SUPPLEMENTARY FIGURE CAPTION**

**Fig. S1 – Differential Expression Profile Analysis of ODAD vs CPF.** The CPF group exhibited a unique DE profile compared to ODAD, evident in both well-defined clusters in PCA (A) and heatmap (B). A total of 30,617 differentially expressed mRNAs were identified. After filtering for genes with a fold change of ≥ ±1.5 and an adjusted p-value of ≤ 0.05, 1,853 genes were found to be downregulated, and 1,884 genes were upregulated. Among them, waterfall plot displays the top 20 upregulated and downregulated genes between the CPF and ODAD groups, highlighting key genes (C). The volcano plot highlights differentially expressed genes between CPF and ODAD groups, with the top 20 genes (D) and the WP Lung Fibrosis (GSEA systematic name M39477) (E) emphasized. Red dots indicate genes within the p-value and fold-change cutoff, blue dots indicate genes above the p-value cutoff, green dots indicate genes above the fold-change cutoff, and green dots indicate genes not significantly different. The dotted lines represent the thresholds: p value cutoff < 0.01, fold change cutoff < 1.5.

**Fig. S2 –** **Differential Expression Profile Analysis of CPF vs f-ILD.** A discrete clustering with some overlap between CPF and f-ILD was noted in PCA (A). The heatmap emphasizes the overlap of cases between the two groups based on all gene expression (B). A total of 31,864 differentially expressed mRNAs were identified, but only 46 and 32 were respectively downregulated and upregulated based on the screening criteria of fold change of ≥ ±1.5 and an adjusted p-value of ≤ 0.05. Waterfall plot displays the top 20 up-regulated and down-regulated genes between CPF and f-ILD groups, highlighting key genes (C). The volcano plot displays the differentially expressed genes between CPF vs. f-ILD, highlighting the top 20 genes most regulated (D) and the genes involved in lung fibrosis as described in WP Lung Fibrosis (GSEA systematic name M39477) (E). Red dots indicate genes within the p-value and fold-change cutoff, blue dots indicate genes above the p-value cutoff, green dots indicate genes above the fold-change cutoff, and green dots indicate genes not significantly different. The dotted lines represent the thresholds: p value cutoff < 0.01, fold change cutoff < 1.5.

**Fig. S3 –Transcriptomic profile of ODAD vs f-ILD.** A substantial overlap between ODAD and f-ILD was also observed in PCA (A) and heatmap (B). Out of a total of 31,367 differentially expressed mRNAs identified, only 3 genes were upregulated and 13 were downregulated, based on the screening criteria of fold change of ≥ ±1.5 and an adjusted p-value of ≤ 0.05. Waterfall plot displays the top 20 up-regulated and down-regulated genes between ODAD and f-ILD groups, highlighting key genes (C). The volcano plot highlight the differentially expressed genes between ODAD vs. f-ILD, focousing on the top 20 genes (D), along with the WP Lung Fibrosis (GSEA systematic name M39477) (E). Red dots indicate genes within the p-value and fold-change cutoff, blue dots indicate genes above the p-value cutoff, green dots indicate genes above the fold-change cutoff, and green dots indicate genes not significantly different. The dotted lines represent the thresholds: p value cutoff < 0.01, fold change cutoff < 1.5.

**Fig. S4 – Normalized gene expression of fibrosis-related and progression-related genes**. The normalized gene expression of MUC5ac (A-B) and WNT10a (C-D), both fibrosis-related genes, shows significantly higher expression (p < 0.05) in CPF and f-ILD compared to CTR. Among the 31 progression-related genes, the normalized expression plots for MUC4 (E-G) and KRT5 (H-J) show higher expression in fibrotic patients compared to their respective controls, with a significant difference (p < 0.05) observed in both CPF vs. CTR and f-ILD vs. CTR.

**REFERENCES**

1 Batah, S. S. *et al.* COVID-19 bimodal clinical and pathological phenotypes. *Clin Transl Med* **12**, e648, doi:10.1002/ctm2.648 (2022).

2 Pathologists, T. R. C. o. RCPath Briefing on COVID-19 – autopsy practice – February 2020. *The Royal College of Pathologists* (2020).

3 Baldi, B. G. *et al.* Clinical, radiological, and transbronchial biopsy findings in patients with long COVID-19: a case series. *J Bras Pneumol* **48**, e20210438, doi:10.36416/1806-3756/e20210438 (2022).

4 Raghu, G. *et al.* Idiopathic Pulmonary Fibrosis (an Update) and Progressive Pulmonary Fibrosis in Adults: An Official ATS/ERS/JRS/ALAT Clinical Practice Guideline. *Am J Respir Crit Care Med* **205**, e18-e47, doi:10.1164/rccm.202202-0399ST (2022).

5 Chen, E. Y. *et al.* Enrichr: interactive and collaborative HTML5 gene list enrichment analysis tool. *BMC Bioinformatics* **14**, 128, doi:10.1186/1471-2105-14-128 (2013).

6 Kuleshov, M. V. *et al.* Enrichr: a comprehensive gene set enrichment analysis web server 2016 update. *Nucleic Acids Res* **44**, W90-97, doi:10.1093/nar/gkw377 (2016).

7 Xie, Z. *et al.* Gene Set Knowledge Discovery with Enrichr. *Curr Protoc* **1**, e90, doi:10.1002/cpz1.90 (2021).

8 Hsia, C. C., Hyde, D. M., Ochs, M., Weibel, E. R. & Structure, A. E. J. T. F. o. Q. A. o. L. An official research policy statement of the American Thoracic Society/European Respiratory Society: standards for quantitative assessment of lung structure. *Am J Respir Crit Care Med* **181**, 394-418, doi:10.1164/rccm.200809-1522ST (2010).
