# Supplementary figures and images for: Transcriptomic profiling reveals the dynamics of fibrotic progression‐related gene expression into post‐coronavirus disease 2019 pulmonary fibrosis
Source: Clin Transl Med. 2024 Nov 13;14(11):e70088. doi: 10.1002/ctm2.70088 (PMC11560857; doi:10.1002/ctm2.70088)

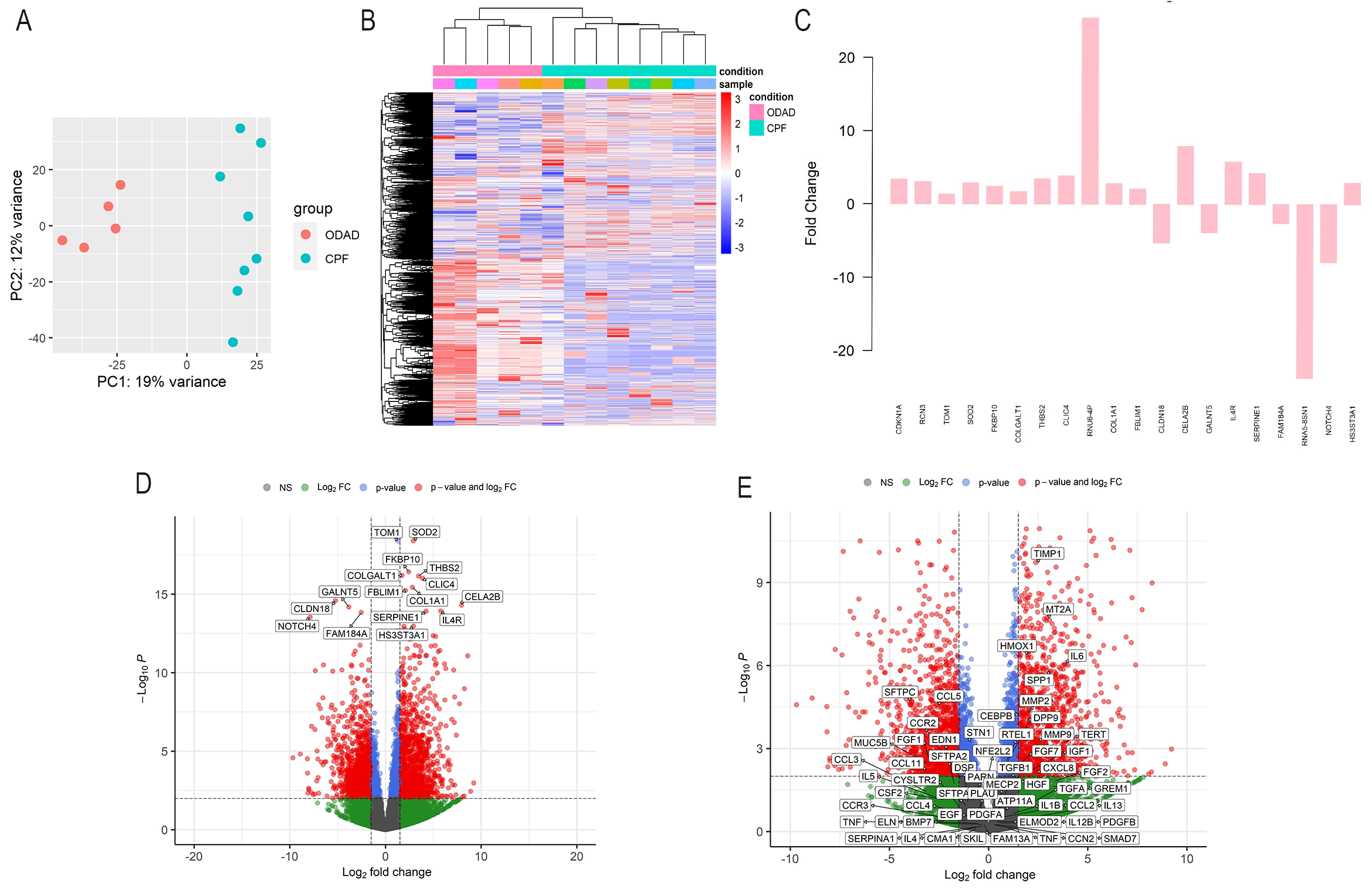

Supplement: Supplementary file 2 — Supporting Information [file CTM2-14-e70088-s002.tif]

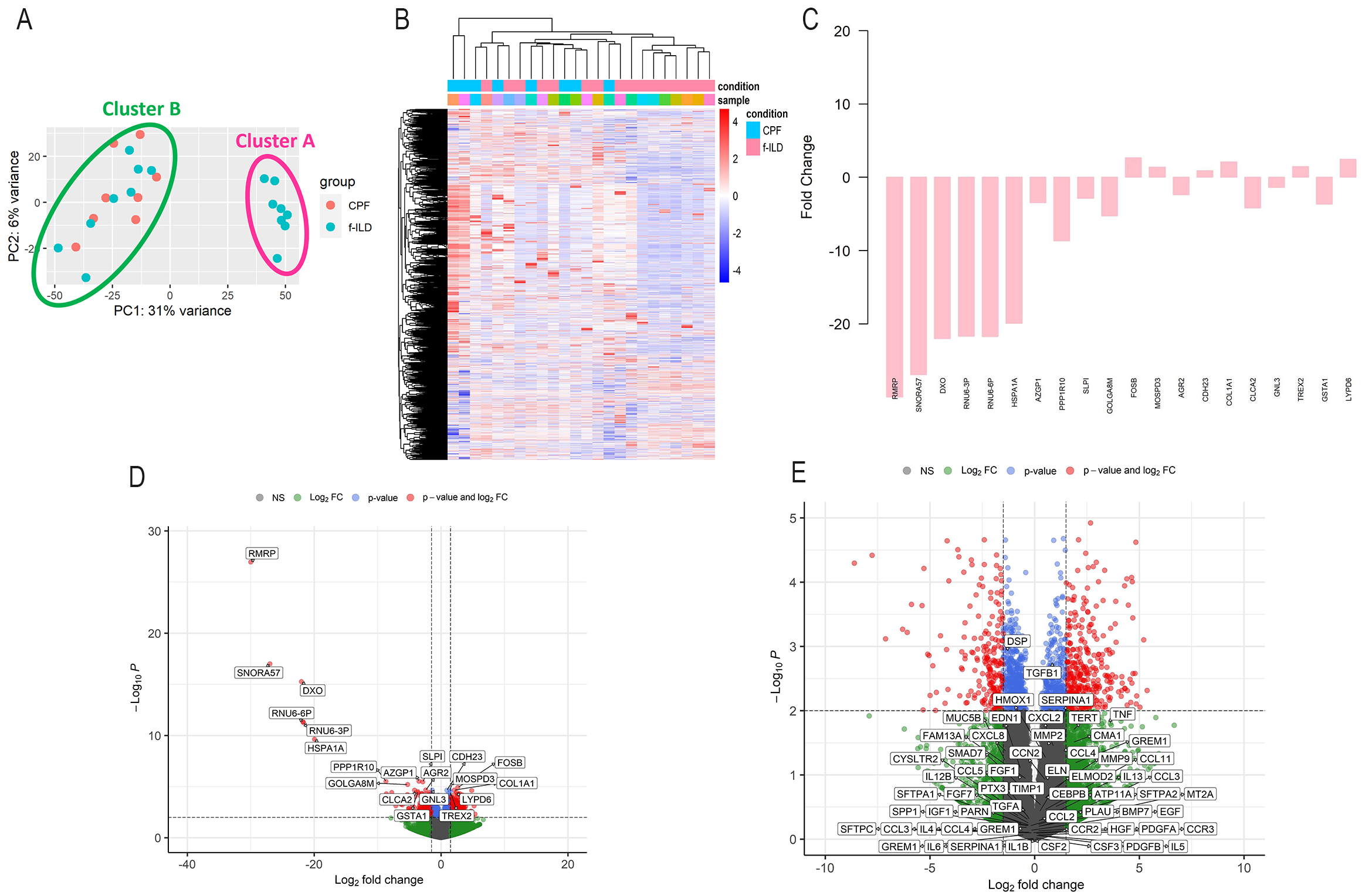

Supplement: Supplementary file 3 — Supporting Information [file CTM2-14-e70088-s003.tif]

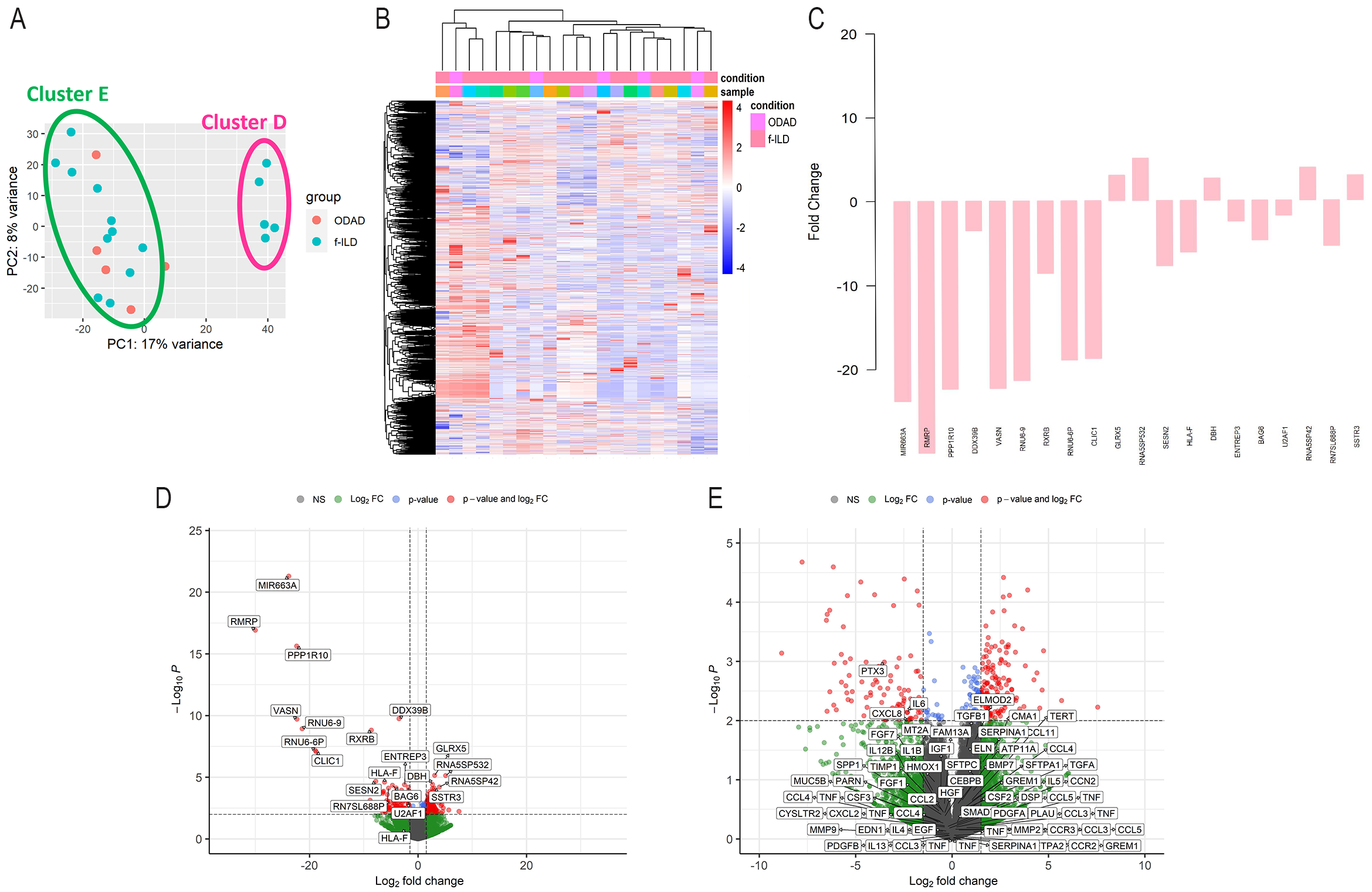

Supplement: Supplementary file 4 — Supporting Information [file CTM2-14-e70088-s005.tif]

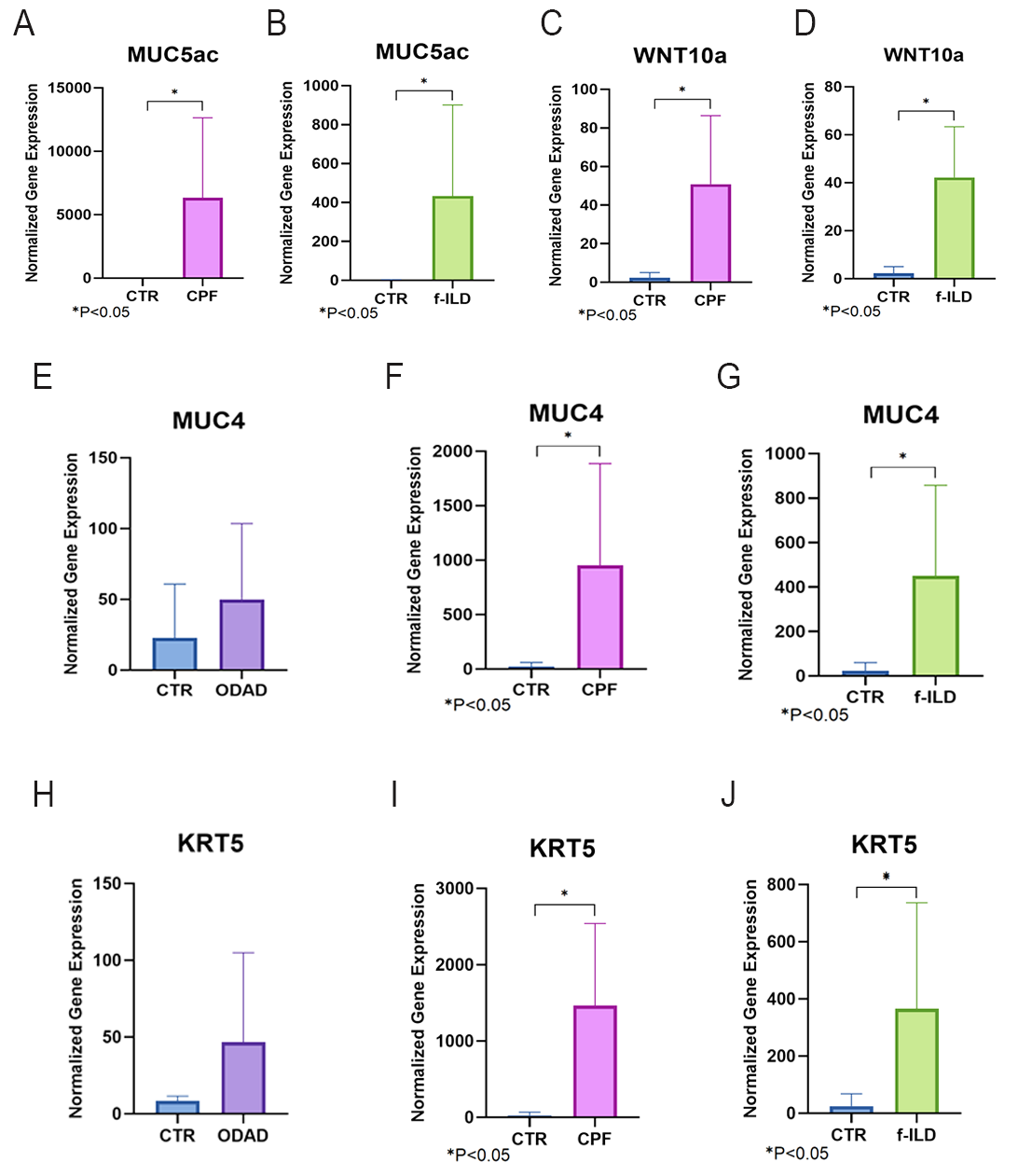

Supplement: Supplementary file 5 — Supporting Information [file CTM2-14-e70088-s001.tif]
